# Polarization in Nursing—Interview Study with Nurse Leaders and Nurses

**DOI:** 10.1177/23779608261421735

**Published:** 2026-03-25

**Authors:** Venla Karikumpu, Kaisa Wikström, Anu Nurmeksela, Marja Hult

**Affiliations:** 1 Department of Sustainable Well-Being, 5475 South-Eastern Finland University of Applied Sciences, Mikkeli, Finland; 2 Department of Nursing Science, 163043 University of Eastern Finland, Kuopio, Finland; 3 Department of Nursing Science, 8058 University of Turku, Turku, Finland

**Keywords:** nursing, nursing leadership, polarization, qualitative study, workplace well-being

## Abstract

**Background:**

Polarization, defined as the division of a group into two distinct and opposing sides, has become increasingly evident in nursing. This phenomenon may negatively affect staff well-being and create significant challenges for nursing management.

**Aim:**

To explore nurse leaders’ and nurses’ experiences and definitions of polarization in nursing practice.

**Design:**

A qualitative study using thematic interviews analyzed through inductive content analysis.

**Participants and Context:**

Data were collected in 2023 from nurse leaders (*n* = 17) through focus group interviews and nurses (*n* = 26) through individual interviews.

**Ethical Considerations:**

The study adhered to ethical research principles. Institutional Review Board (IRB) approval was obtained from the relevant trade union prior to data collection. All participants provided informed consent.

**Findings:**

Both nurse leaders and nurses perceived nursing as polarized and deeply embedded in everyday practice. Polarization manifested through diverging individuality, siloed work units, organizational structures, and pressing societal challenges. Diverging individuality was linked to personal preparedness and demographic factors, while siloed work units reflected divisions within work communities. Organizational structures were shaped by sector-specific characteristics and employment relationships. Societal challenges included differing positions on multiculturalism, geographical disparities, and other contextual factors.

**Conclusions:**

Polarization in nursing emerges from individual, organizational, and societal dimensions. Nurse leaders primarily interpret polarization through organizational challenges, such as role conflicts and work community dynamics, whereas nurses experience it more at the individual level, for example, through reward systems. Addressing both perspectives is essential for identifying, mitigating, and preventing polarization. Understanding its multidimensional nature enables nurse leaders to implement strategies that foster inclusivity and collaboration, ultimately enhancing staff well-being, improving patient care, and strengthening organizational performance.

In contemporary scholarly and public debates, polarization is increasingly conceptualized as the segmentation of societal groups along divergent ideological lines, a process amplified by the algorithmic dynamics of social media platforms (Jacob & Banisch, 2023; Villarruel & Gennaro, 2023). Beyond ideological divides, polarization also manifests in labor markets through job polarization, a phenomenon driven by technological change that disproportionately affects occupational categories. This structural shift results in the concurrent growth of low-skilled, low-wage routine jobs and high-skilled, high-wage positions (Autor & Dorn, 2009; Fernández-Macías, 2012). Despite its relevance, both ideological and job polarization remain underexplored in nursing. Emerging evidence suggests a widening gap between high-status professionals and an expanding segment of low-paid, precarious workers (Gül et al., 2023), reflecting gendered labor divisions and economic vulnerability. These disparities are further compounded by poor working conditions, limited career progression, and inadequate remuneration, factors that contribute to workforce instability and high turnover rates in the healthcare sector. Such division of healthcare workforce increase inequalities, often intersected alongside gender, by characteristics such as age, ethnicity or migration status, education and occupational status (Graells-Sans et al., 2025). Consequently, current trends raise critical concerns regarding the sustainability of a skilled workforce capable of delivering quality care (Shen et al., 2024). Understanding the drivers, manifestations, and consequences of polarization and inequality in nursing is essential for designing effective interventions and policies that promote equity and high-quality care in healthcare organizations.

## Literature Review

Polarization is defined by the clustering of individuals or groups at the extremes of a distribution, such as income, occupational status, or health, resulting in a shrinking middle and growing divides between high- and low-status groups (Fusco & Silber, 2014; Kurer & Palier, 2019). Job polarization and ideological polarization represent distinct yet interrelated forms of societal ramification, each contributing to a divergence within the economic and political landscapes.

Job polarization has emerged as a critical area of inquiry in labor economics, characterized by a structural shift in employment patterns. This polarization occurs as job opportunities expand at the high-skill, high-wage end, and low-skill, low-wage sectors, while middle-skill jobs face decline. Research indicates that technological advancements and globalization are primary drivers of this phenomenon. Automation and offshoring have notably displaced routine jobs, particularly in clerical, manufacturing, and certain service sectors, leading to a hollowing-out effect (Canon & Liu, 2014; Naticchioni et al., 2014). Studies demonstrate that, over the last few decades, demand for middle-skill jobs has diminished, increasing the share of employment in high-skill professions like engineering and low-wage roles in caregiving and services, thereby increasing income inequality and reducing economic mobility (Canon & Liu, 2014; Naticchioni et al., 2014). Moreover, the routinization hypothesis supports the argument that technological changes tend to favor high- and low-skill occupations, reinforcing the trends of job polarization (Naticchioni et al., 2014). The consequences of this division are significant: not only does it contribute to wage inequality, but it also intensifies the challenges associated with maintaining a robust middle class, thereby amplifying societal disparities (Canon & Liu, 2014). This dynamic fosters a growing economic divide that has sociopolitical consequences.

Conversely, ideological polarization focuses on the widening ideological rift between political groups. This phenomenon encompasses both ideological polarization, which involves divergence in policy preferences along a left–right continuum, and affective polarization, characterized by increased animosity and distrust toward opposing groups (Iyengar et al., 2019; Wilson et al., 2020). Political scientists highlight that as party systems, especially in two-party contexts, become more polarized, there is a significant decrease in ideological overlap among party elites and the general populace. This trend undermines the middle ground necessary for effective democratic deliberation and compromise (Hernández et al., 2021; Torcal & Magalhães, 2022). The interplay between growing ideological coherence among political elites and the fragmentation of public opinion underscores how affective polarization can transform political identities into sources of societal division. Individuals increasingly view political adversaries as existential threats, leading to distrust and a more polarized public square where compromise becomes increasingly difficult (Lu & Lee, 2018; McCoy & Somer, 2018). In summary, job polarization and ideological polarization together illustrate a broader trend of societal division, wherein structural and behavioral transformations in labor markets echo and exacerbate political divisions, severely impacting democratic governance and social equity (Hare, 2021; McCoy & Somer, 2018).

Very few studies apply the concept of polarization to nursing, but those that do demonstrate how increasing societal polarization shapes nursing's professional identity, autonomy, and relational dynamics. de Araújo et al. (2021) reveal polarization as a manipulative legal discourse in Brazil, where medical elites construct an “us versus them” narrative to delegitimize nurses’ autonomy in primary care, using exaggeration, evidentiality, and war metaphors to frame nursing as incompetent and dangerous. Similarly, Perry et al. (2024) acknowledge political polarization as a structural barrier to health policy engagement among doctoral nursing students. Morley et al. (2023) in turn, document polarization at the community level during COVID-19, where politicization of public health measures fractured trust, intensified isolation, and undermined nurses’ sense of belonging, despite pockets of solidarity within teams. Last, Noth-Matchett and Stoerger (2025) shift the lens inward, addressing workplace polarization and social outrage as threats to team cohesion and professional identity, advocating for psychological safety and authentic dialogue. Collectively, these studies underscore that polarization operates across legal, political, organizational, and societal domains, both as an instrument of power and as a cultural challenge, requiring strategies that integrate structural advocacy, ethical leadership, and relational resilience to safeguard nursing's autonomy and cohesion.

Several studies, however, describe conditions in nursing workplaces that can be associated with polarization; one example is the technology-driven polarization of work (Wu et al., 2022). Nursing combines routine and non-routine tasks, encompassing both manual and cognitive elements. Polarization tends to increase cognitive inflexibility, meaning individuals become less capable of updating their beliefs or considering alternative perspectives. This rigidity can reinforce group cohesion and deepen polarization (Wu et al., 2022). Cognitive inflexibility may hinder open dialogue and mutual understanding in nursing workplaces, creating morally distressing situations for nurses. However, opposing views may also reflect value differences, which correspond to ethical conflicts among individuals or between individuals and organizations in nursing settings (Rainer et al., 2018). Moreover, highly technology-intensive services (e.g., acute care and surgery) are better resourced and require nursing professionals with specialized training to manage complex cognitive tasks. In contrast, services such as elderly care remain labor-intensive. Elderly care has often been subject to neoliberal market forces, resulting in lower wages and increased precarity in the sector (Fité-Serra et al., 2019). Also, educational differences divide nurses through role advancement (Scott & Cleary, 2007). Workplace polarization negatively impacts nurses’ well-being, contributing to burnout when they experience a lack of support and disengagement (Kamath et al., 2025), which in turn hinders nursing leadership. Therefore, nurse leaders who focus on managing workplace polarization enhance their ability to recognize and balance opposing poles in nursing practice (Noth-Matchett & Stoerger, 2025; Sorour et al., 2023).

Given the growing importance and limited understanding of polarization in nursing, this study explores how nurse leaders and nurses experience and describe polarization. We focus on ideological and job-related polarization because both significantly affect patient care and the nursing workforce. Although ideological polarization often refers to political opinions, it can also manifest in personal beliefs, such as trust in scientific evidence. Nursing education and practice are evidence-based; however, an increasing number of individuals rely on alternative views on care and health. Ideological polarization in nursing is evident in attitudes toward issues such as abortion (Alspaugh et al., 2022) and childhood vaccination (Deem et al., 2020).

### Research Aim

This study aimed to explore nurse leaders’ and nurses’ experiences and conceptualizations of polarization in nursing practice. The research question guiding the study was: How do nurse leaders and nurses describe the manifestation of polarization in nursing?

## Methods

### Research Design

A qualitative semi-structured interview study was conducted using data triangulation and was reported according to the Standards for Reporting Qualitative Research (SRQR) guideline (O’Brien et al., 2014). Qualitative study design was used because the topic is very sparsely studied in professional nursing.

### Participants and Research Context

This study was conducted among Finnish registered nurses and nurse leaders. Participants were recruited from volunteers who had responded to a 2023 survey on employment quality, distributed through the national trade union for social and healthcare workers. A random sample of 50 nurses was invited, and 26 volunteers participated in individual interviews conducted by one researcher in June 2023. Additionally, 68 nurse leaders volunteered for interviews, and ultimately 17 participated. Three researchers conducted six focus groups with nurse leaders in autumn 2023, each comprising one to five participants. The mean duration of the nurses’ interviews was 47 min, while the focus groups with nurse leaders averaged 98.

Background information (Table 1) collected from both participant groups included age, gender, and profession. Additionally, nurses were asked about their years of work experience, while nurse leaders provided their highest level of education. The respondents represented a broad geographic distribution across Finland and covered a wide range of specialties, including both primary and specialized healthcare. The interview guide (see Supplemental Material 1) was developed collaboratively by the research team, drawing on insights from previous literature (Kallio et al., 2016). With participants’ consent, all interviews were audio-recorded and subsequently transcribed verbatim.

**Table 1.** Participants’ (*n *= 43) Background Information.

	Leaders (*n* = 17)		Nurses (*n* = 26)	
	n	%	n	%
Education in nursing				
Professional degree	1		–	–
Advanced practice nursing	1	6%	–	–
Bachelor's degree	10	59%	–	–
Master's degree	5	29%	–	–
Profession				
Dental nurse	–	-–	2	8%
Paramedic	–	-–	1	4%
Practical nurse	–	–	4	15%
Public health nurse	–	–	1	4%
Registered nurse	–	–	18	69%
Assistant head nurse	7	41%	–	–
Head nurse	7	41%	–	–
Chief nurse	2	12%	–	–
Nurse director	1	6%	–	–
Gender				
Women	15	88%	23	88%
Men	2	12%	2	8%
Other	–	–	1	4%
	*Mean*	*Median*	*Mean*	*Median*
Age (leaders: ranging from 36 to 61 years, nurses: ranging from 26 to 62 years)	52	55	47	48
Work experience (ranging from 5 to 40 years)	–	–	20	20

### Data Analysis

The data were analyzed using inductive content analysis (Hsieh & Shannon, 2005). The nurse leader dataset contained 350 original expressions, and the nurse dataset 505. Data saturation was reached at the 21st nurse interview, and all volunteer leaders were interviewed. Two researchers independently analyzed anonymized nurse and nurse leader data. They first read the transcripts repeatedly to gain an overall understanding, then extracted relevant content based on a mutually agreed analysis unit, one or more sentences answering the research question. Original expressions were condensed and grouped into preliminary subcategories by similarity. One researcher processed leader data in Atlas.ti, and the other processed nurse data in MS Word. Subcategories from both datasets were then combined into a shared file for comparison. After independent and collaborative review, consensus was reached, and subcategories were uniformly named and abstracted into broader categories based on thematic similarities. During the integration of sub-datasets and the final stage of analysis, the entire research team reviewed and discussed the results to reach consensus. The two primary analysts reported the findings according to four main categories, illustrating each with original expressions from both datasets to support the descriptions.

### Ethical Considerations

The study adhered to established ethical research principles (ALLEA, 2025). Approval was obtained from the Institutional Review Board (IRB) of the trade union. Participants received an information sheet prior to the interview and provided informed verbal consent before participation. As interviews were conducted remotely, consent was audio-recorded.

## Findings

**Figure 1.**
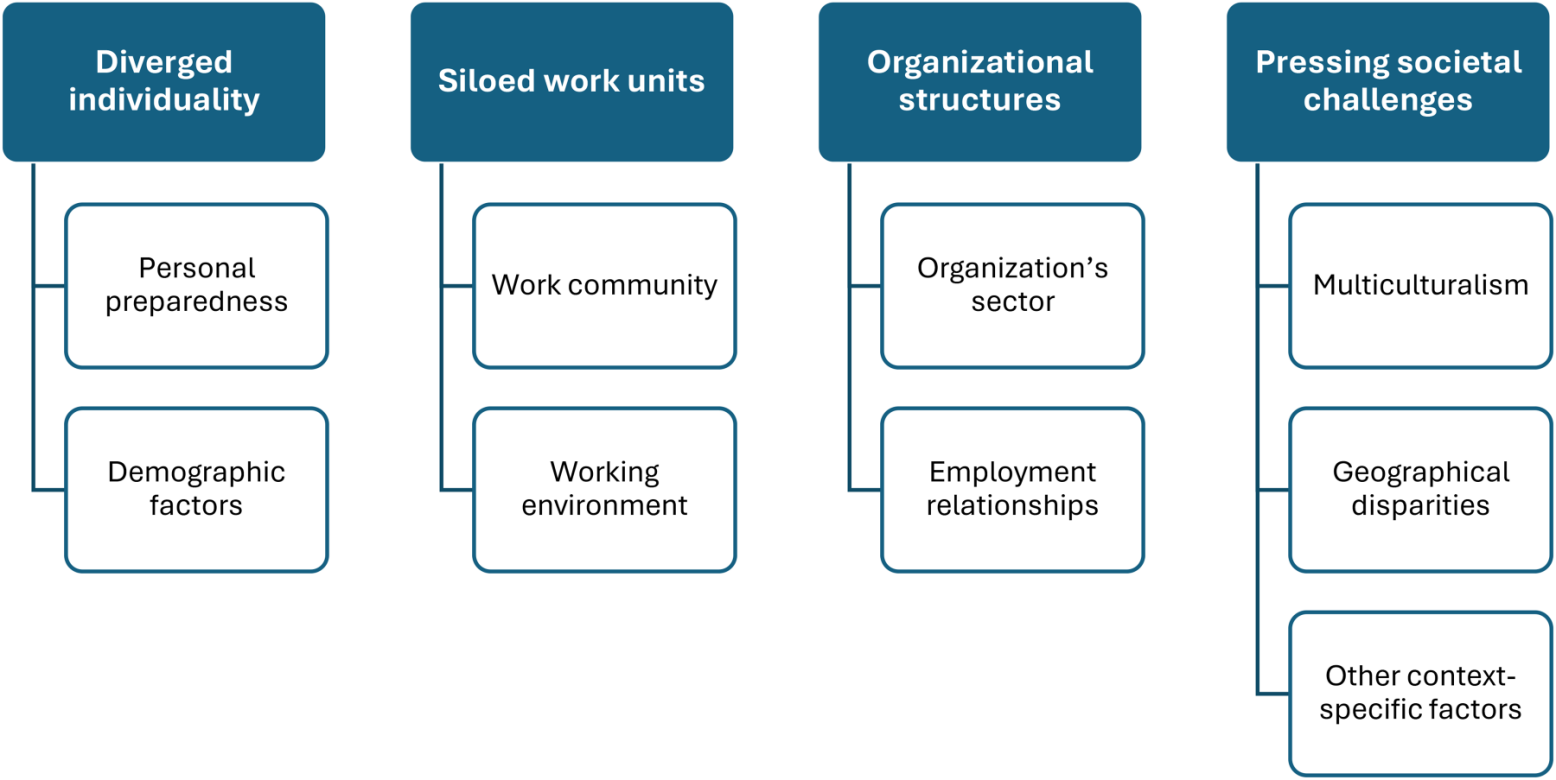
Participants’ perspectives on polarization in nursing.

Polarization was categorized into four main categories based on interviews with nurse leaders and nurses: diverged individuality, siloed work units, organizational structures, and pressing societal challenges (Figure 1).

### Diverged Individuality

Polarization within the main category of diverged individuality was further classified into *personal preparedness* and *demographic factors*. Personal preparedness encompassed differences in individuality, scientific attitudes, and work ability. Nurse leaders acknowledged nurses’ diverse life situations, describing them as a polarizing factor linked to exhaustion. Both groups identified jealousy among nurses, particularly between age groups and between temporary and permanent employees. Leaders reported value differences that influenced compliance with organizational guidelines, whereas nurses emphasized variations in working methods. Some nurses noted that peers imposed self-selected constraints and task preferences, creating conflict. Leaders, however, perceived experienced nurses as more flexible compared to younger nurses, who were seen as having greater demands. One leader illustrated this by stating:

A certain kind of selfishness is prevalent today, so we can no longer discuss and agree on work shifts as we used to. (Group 2, Leader 1)

According to nurse leaders, a scientific attitude was regarded as a positive trait, whereas nurses reported the opposite experience. Leaders believed that nurses enter the profession with an interest in evidence-based practice, but noted that exposure to disinformation increased during fieldwork. Nurses, in contrast, perceived that many peers did not consider nursing science a necessary foundation for practice, as illustrated by the following simplified expression:

It's thought that nursing work is more like something where science isn’t really needed. I’ve definitely noticed that attitude a lot. (Nurse 3, Female)

From the perspective of both professional groups, differences in work ability became evident in relation to working hours, as reduced work ability increasingly led some employees to choose part-time positions. Both groups noted that part-time work placed additional strain on the work community. Nurses reported that when leaders reduced workloads for certain employees, the burden on others increased, complicating shift and task distribution. One nurse described these challenges as follows:

They can’t be assigned to the evening shift, because it puts more strain on them if they have any musculoskeletal issues, so it just doesn’t work like that. (N18F)

Demographic factors included differences in age, gender, and competence. Both professional groups noted that age, combined with career stage, clearly divided nurses within work communities. Leaders observed a shift in work ethic among younger nurses, reflected in decreased self-direction, lower quality goals, and, in the view of both groups, reduced responsibility-taking. Leaders described young nurses as doing only the bare minimum, while nurses reported that younger colleagues were selective about tasks, sometimes refusing those they did not prefer. Younger nurses were also perceived as highly aware of their rights but lacking work-life skills. Conversely, they faced unfair criticism and were often required to prove their competence. Despite these challenges, both groups characterized young nurses as enthusiastic and inclined to question established practices.

Both professional groups emphasized that younger nurses appeared less committed compared to previous generations. Leaders reported that highly committed nurses were typically over 45 years old. This generational difference was perceived as hindering work unit development and, in some cases, bringing it to a halt. Nurses described older colleagues as demanding, inflexible, resistant to change, and reluctant to question established practices. According to both groups, these traits made it difficult for new nurses to integrate into committed work communities. One nurse illustrated the impact of age as follows:

So, when new employees come in who might question things more, and their way of thinking is a bit different, you can sometimes notice that older employees might be a bit hesitant or resistant to that. (N3F)

According to nurse leaders, gender appeared to contribute to increased cliquishness within female-dominated work units. Nurses also described this phenomenon, noting that workplace practices could become more rigid and occasionally escalate. Female nurses were perceived as seeking groups that shared their personal ideology, as illustrated by one nurse in the following statement:

[Women are] looking for that connection, the kind where you can talk badly about the employer, supervisor, or others together. (N10F)

According to nurse leaders, differences in competence were increasingly perceived as a positive attitude toward further education, often seen as a pathway out of fieldwork, which contributed to shortages of specialized nurses in units. Nurses, on the other hand, described differentiation through competence gaps and variations in educational backgrounds. Competence gaps, such as lacking authorization to administer medication, were reported by both groups as placing strain on workplaces. Nurses also highlighted disparities in competence and perceived value based on education, particularly between practical nurses and registered nurses, vocational college and university of applied sciences degrees, and between master's degrees in applied sciences and university-level master's degrees. Frequent comparisons between these qualifications were noted, as illustrated by one nurse in the following statement:

The fact that a master's degree in applied sciences doesn’t result in a salary increase, but someone who has completed the right to prescribe medication does, causes frustration, with the question for what good reason? (N21F)

### Siloed Work Units

Polarization within siloed work units was categorized into two dimensions: the *work community* and the *working environment*. The work community was divided by leadership actions and positions within the team. According to leaders, nurses with varying levels of experience increasingly required different approaches to management. Consequently, nurses perceived that leaders favored certain individuals by granting them more freedom and rights. Leaders acknowledged that individualized management posed challenges to ensuring equal treatment. Nurses emphasized that leadership was essential for leveraging and sharing competence and for modeling values and attitudes. One leader described the necessary leadership actions as follows:

Young employees who have entered this field and feel passionate about nursing should not be exhausted during their first years of work. (G3L1)

Both professional groups acknowledged divisions based on positions within the work community. Nurses’ job descriptions were characterized as versatile, as they could work across multiple specialties and in multiprofessional teams, either as one of several experts or as the sole expert. According to both groups, nurses working as the only nursing professional were perceived as having their expertise stand out more clearly than those working within a team of nurses. Furthermore, nurses noted that registered nurses were often allowed to assume managerial roles in units where other staff had lower-level training. However, nurses with managerial responsibilities reported that their administrative work was not recognized as “real work” by other staff members, as illustrated by the following statement:

So sometimes it gets forgotten that, since I’m not working much at the grassroots level, the question of what my position is there and what I do might not be clear. (N18F)

The working environment varied across specialties and units. Both professional groups noted that certain units and specialties attracted and retained staff due to their specialized competence requirements, while immigrant employees faced additional boundaries. Both groups expressed concern that workers with limited language skills—considered harder to employ—tended to concentrate in less appealing specialties, such as elderly care. Additionally, nurses reported rivalry between specialties, as illustrated by the following statement:

Well, it probably shows in a way that places like acute care, intensive care, those are kind of trendy workplaces, so maybe it gives the feeling that when a person works in such a place, it's like wow. (N17F)

### Organizational Structures

Polarization within organizational structures was categorized by the *organization's sector* and *employment relationships*. The sector was described in terms of service level and organizational characteristics. From leaders’ perspectives, divisions were evident in the greater appreciation of specialized healthcare compared to primary healthcare. Nurses reported higher competence and language requirements in specialized healthcare, while leaders viewed competence in primary care as broader but less complex. Nurses also noted that work pace was faster in primary care, whereas specialized care had better resources. Consequently, leaders associated higher turnover rates with primary care. Additionally, nurses perceived process development as more efficient in primary care due to the faster implementation of changes. The perceived appreciation of specialized healthcare is reflected in the following statement from one leader:

The status in specialized healthcare has been thought that there you are held with better hands, thus also part of the patients are asking for a referral to get into the outpatient clinic. (G1L4)

According to leaders, private social and healthcare services were generally perceived as more insulated from polarization. However, this was also seen as deepening the gap between the private and public sectors. Nurses reported being more aware of the costs of care and procedures in the private sector. Both groups highlighted stronger retention power in private organizations compared to public ones, as reflected in the following statement from a leader:

[In the private sector…] people are working for us, and usually they don’t have any other employer. (G1L1)

The employment relationship was categorized into terms of employment and rewards. Regarding employment conditions, leaders observed a declining attitude toward shift work and an increased willingness among nurses to accept temporary positions. Nurses reported that labor shortages had fostered a more positive attitude toward temporary workers. Both groups noted that temporary employees often lost workplace benefits, such as coverage for medical expenses, strike compensation, and training opportunities. Nurses emphasized that because temporary workers typically performed only basic tasks, responsibility for broader duties fell on permanent staff. Leaders shared this concern and suggested that more comprehensive integration of temporary workers into daily unit operations could reduce polarization caused by workload disparities. One nurse described this perceived injustice as follows:

Fixed-term employees still don’t have the same rights and possibilities as the permanent employees do. (N7F)

Leaders perceived reward-related differences in the attitudes of newly graduated nurses, who often regarded salary as a secondary concern. Conversely, nurses expressed dissatisfaction and a sense of inequality regarding wage disparities linked to educational differences, which they considered unjustified. They also reported salary fluctuations within units, sometimes followed by inappropriate behavior, as illustrated by the following statement:

I’ve heard that in many places, summer substitutes are treated poorly, because their salary is higher. (N23F)

### Pressing Societal Challenges

Polarization within topical societal challenges was categorized into *multiculturalism*, *geographical location*, and other *context-specific factors*. Multiculturalism was further divided into attitudes and language. From leaders’ perspectives, workplace attitudes toward increased work-related immigration were often negative, with some reports of racism. Leaders associated this with a diminished sense of community within workplaces. Conversely, nurses described international colleagues’ attitudes as positive and characterized employees from diverse ethnic backgrounds as hardworking and adaptable. One nurse expressed this view as follows:

It should be understood that they [international nurses] can have know-how, they can be really good employees, but have they managed to integrate into this society yet properly? (N5F)

Leaders and nurses identified language skills as the primary source of polarization among international nurses; those with stronger language proficiency integrated more effectively into work communities. Nurses reported that limited language skills created hazardous situations and fostered discriminatory attitudes among local staff, while also contributing to the isolation of immigrant nurses. Both groups noted that native-speaking nurses experienced an increased workload burden. Nurses further emphasized that international nurses’ competence could not be fully utilized due to differences in educational qualifications, whereas leaders highlighted challenges related to extended orientation periods. This concern is illustrated in the following statement:

During the night shift, one doesn’t talk much, and the other doesn’t really understand, so it's difficult to, for example, assign patients properly. (G5L1)

Geographical location was not mentioned by leaders, but nurses described employees in rural areas as being subject to stereotypes of weaker competence. Nurses’ job descriptions were considered more limited in urban settings, where multiprofessional experts were readily available for consultation. In contrast, nurses in rural areas perceived their roles as more diverse and reported working more independently. Additionally, nurses in cities experienced greater inequality in living and commuting conditions, as longer commutes were common due to hospitals being located near city centers. This concern is reflected in the following statement:

It's somehow wrong that some have to pay [to get to work], and some get the same things for free, when after all, the salary is the same for everyone. (N12F)

Leaders identified evolving societal factors as contributors to nursing polarization. They noted variations in attitudes toward protective behaviors across units during the COVID-19 pandemic and reported that striking nurses were perceived as having lower status during labor actions. Nurses’ responses reflected concerns about professional recognition and treatment, which they felt remained largely rhetorical in politics and society. Nurses also criticized the media for exaggerating and distorting patient cases without fully understanding clinical realities. Additionally, leaders highlighted labor market shifts favoring nurses over recent decades, which they viewed as increasing organizational challenges—challenges that some organizations addressed through improved attractiveness. This perspective is illustrated in the following statement:

In the past, it might have been said that we won’t give you such a small percentage of working hours unless there is a statutory obligation to reduce working hours. But now, the situation is such that the labor market is so favorable for employees that if you don’t give it, someone else will. (G2L2)

## Discussion

This study provides novel insights into how polarization manifests in nursing, as experienced by both nurse leaders and staff nurses. Growing polarization within the profession appears across individual, unit-level, organizational, and societal dimensions, shaped by the distinct roles of leaders and nurses. Nurse leaders tended to frame polarization primarily through an organizational lens, emphasizing role diversity and challenges within the working community. In contrast, nurses’ accounts were rooted in everyday practice and observations, incorporating intersectional factors such as gender, age, ethnicity, and remuneration. Despite these differences in emphasis, both groups perceived polarization as sharing similar underlying attributes across all levels.

The challenges faced in nursing mirror those encountered in other sectors of society. Among these, issues related to the internationalization of the nursing workforce were reported most frequently. Our findings reveal negative attitudes, prejudice, and even instances of racism toward internationally recruited nurses. The most significant barrier to successful internationalization was linguistic. Consistent with previous research, limited proficiency in the local language not only posed patient safety risks but also contributed to discriminatory attitudes among local staff and reinforced the segregation of international nurses. Shiju et al. (2024) argue that language difficulties negatively affect patient care and increase anxiety and insecurity among international nurses. Similarly, our focus group discussions indicated that international nurses often occupy positions below their qualifications due to skill gaps or differences in educational backgrounds. Studies highlight that inadequate policies can hinder international nurses from utilizing their full potential and integrating into workplace communities (Xiao et al., 2014). As the nursing workforce becomes increasingly global, standardized practices and sustained commitment are required at all organizational levels. While internationalization offers clear benefits for healthcare organizations and teams, it demands coordinated efforts from individuals, work communities, organizations, and societies.

This study underscores the pivotal role of nurse leadership as a guiding force within the work community. Both leaders and staff nurses emphasized the importance of equitable leadership practices to maintain cohesion and foster collaboration. Previous research similarly demonstrates that nurse leaders facilitate progress by addressing, acknowledging, and constructively discussing divergent viewpoints (Noth-Matchett & Stoerger, 2025). Leaders who act as role models—demonstrating professionalism, empathy, and transparency—help bridge generational and experiential divides, as authentic leadership builds trust, a cornerstone of effective teamwork (Javidan et al., 2023; Noth-Matchett & Stoerger, 2025). By adopting an early intervention approach and creating space for open dialogue, nurse leaders can proactively manage and resolve conflicts among professionals. Furthermore, nurse leaders serve as key intermediaries, strengthening organizational connections and facilitating collaboration across specialties and units (Noth-Matchett & Stoerger, 2025).

Our findings indicate that age intersects with diminished work capacity, creating challenges for work ability. Previous studies have shown that aging nurses often remain motivated to continue working despite reduced work ability, driven by an intrinsic desire to contribute to society (Nurmeksela et al., 2023; Stimpfel et al., 2020). However, our results reveal that reduced work ability can strain work communities, as accommodations for these individuals, such as modified tasks and limited working hours, often increase the workload for others. In practice, nurses with reduced work ability were restricted to specific shifts, requiring colleagues to cover a greater share of shifts they could not perform. To support aging nurses, tailored interventions such as scheduling shifts aligned with chronotype, reducing working hours, and offering regular weekday office hours have been recommended (Nurmeksela et al., 2023; Stimpfel et al., 2020). Above all, aging nurses perceive leadership and organizational structures as the most critical factors in sustaining their work ability (Stimpfel et al., 2020).

The findings on employment terms align with broader trends, as temporary and part-time work have generally increased across Europe, largely following the global financial crisis of 2008–2010 (Torrejón Pérez et al., 2023). Additionally, the division between specialized and primary healthcare as well as and the higher prestige associated with certain specialties, appears to reflect a technology-driven generation, particularly in specialized care. Consequently, specialties such as elderly primary care, which require more hands-on work, have shown limited engagement in organizational development initiatives. In contrast, process-oriented approaches to healthcare development could help bridge the gap between specialized and primary care, fostering greater integration and collaboration (Poulsen et al., 2025).

Our study suggests that media plays a significant role in shaping the perceived value and recognition of nursing. de Araújo et al. (2021) demonstrate how manipulative discourse can influence power relations and nurses’ societal positioning. Our findings, however, were more pragmatic: media was perceived as guiding young nurses’ specialty choices by promoting acute care settings as prestigious, while portraying less acute areas as less valued. Research on the influence of media and social media on nurses’ specialty selection remains limited, yet this topic warrants further exploration, as it may already affect recruitment patterns, contributing to workforce imbalances across specialties. It is plausible that media, particularly social media, shapes career aspirations among young professionals. Additionally, political decision-making may partly determine the perceived value of different specialties, which could further influence specialty selection and workforce distribution.

### Strengths and Limitations

The strength of this study lies in its rich and diverse dataset, collected from all members of the work community, including both leaders and nurses. To ensure credibility, all phases of the research process were rigorously documented. Trustworthiness was further enhanced by integrating two perspectives, leaders and nurses, into the analysis. Initially, data were analyzed separately for each group; subsequently, the analyses were merged through a coding process that demonstrated reliability by constructing codes from shared themes (Coleman & Ed, 2021). The study adhered to established ethical principles, ensuring that no harm was caused to voluntary participants. The researchers’ backgrounds in nursing and nurse leadership provided a deep understanding of the participants and the context. Moreover, the research team actively reflected on and discussed potential biases throughout the study, maintaining transparency and reflexivity (Coleman & Ed, 2021).

Polarization in nursing remains an underexplored phenomenon. The concept of polarization was deliberately employed rather than alternatives such as inequality, responding to calls for research explicitly addressing polarization in nursing and acknowledging its current relevance (Villarruel & Gennaro, 2023). Polarization in care work reflects broader socio-economic and gendered disparities, highlighting systemic issues that require comprehensive policy responses. During data analysis, participants’ views revealed a pervasive integration of polarization, reinforcing the contextual validity of the findings.

### Implications for Practice

The consequences of polarization and inequality are extensive, impacting not only individual workers but also the quality, safety, and equity of healthcare delivery. Polarization and inequality undermine care quality by eroding team cohesion, increasing burnout, and reducing job satisfaction (Pandey & Chandu, 2025). Workforce maldistribution and turnover increase access gaps, particularly in underserved regions, and compromise patient safety (Bogue & Bogue, 2020). Among nurses, inequality and discrimination are associated with higher rates of burnout, depression, and anxiety, especially among minoritized and female workers (Bafna et al., 2025). Elevated burnout levels, in turn, correlate with increased medical errors, diminished professionalism, and lower patient satisfaction (Pian et al., 2019). Furthermore, exposure to violence and microaggressions further impairs mental health and heightens attrition risk (Stufano et al., 2025). Organizations can mitigate these risks through structured diversity training programs that address microaggressions and unconscious bias, which have proven effective in reducing subtle forms of discrimination (Roberson et al., 2020). Inclusive human resource practices, such as transparent recruitment, promotion processes, and accountability mechanisms, are essential for fostering fairness and reducing exclusion (Gayathri & Savarimuthu, 2025).

## Conclusion

This study offers novel insights into polarization within the nursing profession, highlighting its complex and multidimensional nature shaped by individual, organizational, and societal factors. Polarization manifests through generational divides, competence- and education-based hierarchies, sector-specific valuation, and language and cultural barriers. These dynamics contribute to reduced team cohesion, perceived inequity, uneven workload distribution, and variable attraction and retention across units and specialties. Recognizing these interconnected dimensions provides opportunities to identify, manage, and prevent polarization in nursing workplaces. By addressing these factors, nurse leaders can implement targeted strategies to foster inclusivity and collaboration, thereby enhancing staff well-being, improving patient care, and strengthening organizational performance.

Polarization and inequality among healthcare workers represent deeply entrenched challenges with significant implications for health systems worldwide. Tackling these issues requires a comprehensive, multi-level approach that combines robust measurement, organizational transformation, and policy innovation to build equitable, resilient, and high-performing healthcare workforces. Future research should prioritize the development of valid measures of polarization and examine longitudinal associations with outcomes such as turnover, evidence-based practice adoption, patient safety, and team functioning across diverse contexts.

## Supplemental Material

sj-docx-1-son-10.1177_23779608261421735 - Supplemental material for Polarization in Nursing—Interview Study with Nurse Leaders and Nurses Supplemental material, sj-docx-1-son-10.1177_23779608261421735 for Polarization in Nursing—Interview Study with Nurse Leaders and Nurses by Venla Karikumpu, Kaisa Wikström, Anu Nurmeksela and Marja Hult in SAGE Open Nursing

sj-docx-2-son-10.1177_23779608261421735 - Supplemental material for Polarization in Nursing—Interview Study with Nurse Leaders and Nurses Supplemental material, sj-docx-2-son-10.1177_23779608261421735 for Polarization in Nursing—Interview Study with Nurse Leaders and Nurses by Venla Karikumpu, Kaisa Wikström, Anu Nurmeksela and Marja Hult in SAGE Open Nursing
