# Supplementary material for: Polarization in Nursing—Interview Study with Nurse Leaders and Nurses
Source: SAGE Open Nurs. 2026 Mar 25;12:23779608261421735. doi: 10.1177/23779608261421735 (PMC13018683; doi:10.1177/23779608261421735)
Supplement: sj-docx-1-son-10.1177_23779608261421735 - Supplemental material for Polarization in Nursing—Interview Study with Nurse Leaders and Nurses [file sj-docx-1-son-10.1177_23779608261421735.docx]

**Interview Themes for Leaders**

**1 Nurses' Diverse Backgrounds and Positions as Employees - Work Polarization**

*Working life and employment have been described as becoming polarized, meaning they are dividing into, for example, successful and disadvantaged employees. The reasons for this can include factors such as the employee's education, age, religion, ethnic background, or language skills. For instance, one phenomenon that divides employees into different/unequal positions is the increase in (voluntary) gig work.*

Q1 How are these factors reflected in your workplace?

Q2 What are the consequences of work polarization?

**2 Nurses' Diverse Worldviews, Values, and Opinions - Ideological Polarization**

*Ideological polarization refers to the distancing of people from each other based on opinions or values. In nursing, one differentiating factor has been attitudes towards vaccines or the COVID-19 pandemic.*

Q3 How does this manifest in your workplace?

Q4 What are the consequences of this phenomenon?

----------------------------------------------------------------------------------------------------------------

**Interview Themes for Nurses**

**1 Nurses' Diverse Backgrounds and Positions as Employees**

*Working life has been described as becoming polarized, meaning it is dividing into, for example, successful and disadvantaged employees. The reasons for this can include factors such as the employee's education, age,* *worldview, ethnic background, and work in urban centers or rural areas.*

Q1 How is this reflected in your work as a nurse?

Q2 What causes it, and how can it be avoided?

**2 Nurses' Diverse Worldviews, Values, and Opinions - Ideological Polarization**

*Ideological polarization refers to the distancing of people from each other based on opinions or values. In nursing, one differentiating factor has been attitudes towards vaccines or the COVID-19 pandemic.*

Q3 How does this manifest in your workplace?

Q4 What are the consequences of this phenomenon?
